# Cardiotoxicity from Capecitabine Chemotherapy: Prospective Study of Incidence at Rest and During Physical Exercise

**DOI:** 10.1093/oncolo/oyab035

**Published:** 2022-02-16

**Authors:** Chiara Lestuzzi, Davide Stolfo, Antonino De Paoli, Alberto Banzato, Angela Buonadonna, Ettore Bidoli, Lucia Tartuferi, Elda Viel, Giulia De Angelis, Sara Lonardi, Roberto Innocente, Massimiliano Berretta, Francesca Bergamo, Alessandra Guglielmi, Gianfranco Sinagra, Joerg Herrmann

**Affiliations:** 1 Cardiology Department, ASFO: Azienda Sanitaria Friuli Occidentale, Cardiology and Cardio-Oncology Rehabilitation Service, Aviano (PN), Italy; 2 Cardiology Department, University Hospital of Trieste, Trieste, Italy; 3 Radiation Oncology Department, Centro di Riferimento Oncologico di Aviano (CRO), IRCCS, Aviano (PN), Italy; 4 Cardiology Service, Veneto Institute of Oncology, IRCCS, Padua, Italy; 5 Oncology Department, Centro di Riferimento Oncologico di Aviano (CRO), IR, Aviano (PN), Italy; 6 Epidemiology Unit, Centro di Riferimento Oncologico di Aviano (CRO), IRCCS, Aviano (PN), Italy; 7 Medical Oncology Unit 1, Veneto Institute of Oncology IRCCS, Padua, Italy; 8 Oncology Department, University Hospital of Trieste, Trieste, Italy; 9 Department of Cardiovascular Medicine, Cardio Oncology Clinic, Mayo Clinic, Rochester, MN, USA

**Keywords:** cardiotoxicity, chemotherapy, capecitabine, ischemia, stress test, arrhythmias

## Abstract

**Background:**

Physical activity may increase the risk of cardiotoxicity (myocardial ischemia, major arrhythmias) of 5-Fluorouracil, but this risk has never been investigated for its prodrug capecitabine.

**Patients and Methods:**

One hundred and ninety-two consecutive patients undergoing capecitabine chemotherapy from December 1, 2010 through July 31, 2016 were prospectively evaluated. The baseline evaluation included electrocardiography (ECG) and echocardiography (2DE); a follow-up evaluation, including ECG and exercise stress testing (2DE in case of ECG abnormalities), was done after ≥10 days of treatment. Cardiotoxicity was suspected from ischemic ECG changes, new kinetic abnormalities at 2DE, Lown classification ≥2 ventricular arrhythmia, symptomatic arrhythmias, or positive stress test, and confirmed by a negative stress test after capecitabine washout.

**Results:**

Cardiotoxicity was diagnosed in 32 patients (16.7%): six at rest and 26 during exercise. All 32 patients had ECG abnormalities: ST-segment changes (24 patients), negative T-waves (2) and/or arrhythmias: ventricular arrhythmias (14 cases), supraventricular tachycardia (2), complete heart block (1). Eight patients had typical symptoms, 6 had atypical symptoms, 1 had syncope, 17 (53%) were asymptomatic. Cardiotoxicity was more common in patients with atypical symptoms during daily life (OR = 15.7) and in those on a therapeutic schedule of 5 days/week (OR = 9.44).

**Conclusion:**

Capecitabine cardiotoxicity is frequent, and often elicited by physical effort. Oncologists, cardiologists, and general practitioners should be aware of this risk. Active cardiotoxicity surveillance with ECG (and echocardiogram and/or stress testing in suspected cases) during therapy is recommended.

**Clinical Trials registration number:**

CRO-2010-17.

## Teaser

This article assesses the overall incidence of both resting and exercise-induced cardiotoxicity in patients undergoing capecitabine chemotherapy and identifies factors associated with cardiotoxicity.

**Implications for Practice**

We found frequent (16.7%) but often asymptomatic or presenting with atypical symptoms (thus possibly underestimated in clinical practice) capecitabine cardiotoxicity. Even moderate physical activities may elicit cardiac ischemia and/or ventricular arrhythmias. To reduce the risk of potentially life-threatening events, the patients should be advised to avoid major physical efforts, to refer to the caring physician for any new cardiac symptom, and have an ECG after some days of therapy done. Patients with suspect cardiotoxicity should be evaluated with either echocardiogram, stress test, and/or Holter monitoring. A negative stress test during therapy has a good negative predictive value for cardiotoxicity.

## Introduction

The anticancer compounds fluoropyrimidines (FP) [5-fluorouracil (5FU) and its oral prodrug capecitabine] are widely used in common cancers, as gastrointestinal, breast and head/neck cancers, alone or in combination with other drugs or radiotherapy; they exert the same spectrum of potentially fatal cardiotoxicity.^1-4^ Capecitabine treatments last several weeks as a single course, or 2 weeks every 3 weeks for several courses.^1-3^ Myocardial ischemia is the most common clinical manifestation of capecitabine cardiotoxicity, but left ventricular dysfunction, ventricular arrhythmias (VA), and sudden death have been reported.^5-10^ Yet no proactive screening protocol to identify patients at risk is currently available.

In retrospective studies, rates of clinically evident cardiotoxicity attributable to FP are low.^11,12^ However, in prospective studies the rates of cardiotoxicity can exceed 30%.^13-15^ The prevalence of exercise-induced FP cardiotoxicity is less well documented. In a prospective study of patients on 5FU,^16^ the overall incidence of cardiotoxicity was 10.3%, and in 43% of these cases, it was induced by physical effort. According to the pharmacokinetics and similarities to 5FU toxicity, also exercise-related capecitabine cardiotoxicity is possible, and it has actually already been reported,^17^ but its incidence has never been investigated. The primary goal of this study was to assess the overall incidence of both resting and exercise-induced cardiotoxicity in patients undergoing capecitabine chemotherapy; the secondary goal was to identify factors associated with cardiotoxicity.

## Patients and Methods

We evaluated consecutive patients undergoing capecitabine chemotherapy between December 2010 and July 2016 at our institutions (two cancer hospitals and one general hospital). Inclusion criteria were: the presence of a solid tumor with clinical indication to start capecitabine chemotherapy; age ≥18 years; performance status ≤2 on the Eastern Cooperative Oncology Group scale^18^; normal serum electrolytes, renal and hepatic function; blood hemoglobin ≥10 g/dL. Exclusion criteria were: left ventricular ejection fraction <50%, or severe aortic stenosis at basal two-dimensional echocardiography (2DE); NYHA class >2; inability to perform an exercise stress test (EST); repolarization abnormalities on resting 12-lead electrocardiography (ECG) limiting data interpretation during EST^19^; tachycardia or frequent VA (Lown classification ≥2) at rest.

The study protocol was approved by the Ethics Committee of each study center. All enrolled patients provided written informed consent to the study and to the treatment of personal data.

### Procedures

At study entry, all patients underwent a clinical cardiologic examination including ECG and 2DE. The clinical data abstracted from clinical charts and patient interviews comprised: age, sex, tumor type, family or personal history of ischemic heart disease (IHD), presence of cardiovascular risk factors (CVRF) as smoking (active or past), hypertension, diabetes, hypercholesterolemia,^19^ and previous and all ongoing cardiac and oncologic treatments. Patients with a history of IHD underwent baseline EST while on their anti-ischemic therapy. All patients were provided a diary to record the onset of new symptoms.

A second cardiologic evaluation including ECG and EST was planned after at least 10 days of capecitabine therapy, during the first course of treatment. ECG was performed before this planned visit in patients reporting major symptoms, and 2DE was done in case of ECG abnormalities. Major criteria for diagnosing cardiotoxicity at rest were: signs of cardiac ischemia (≥2 mm ST elevation or horizontal ST-segment depression on ≥3 leads at ECG, new diffuse or segmental kinetic abnormalities on 2DE),^19^ symptomatic arrhythmias, and frequent VA (couplets or more than 10% of all ventricular depolarizations during any 30-second ECG recording) even if asymptomatic. Minor criteria were: typical angina, angina equivalents (dyspnea, atypical chest pain, jaw pain),^19^ new (compared to basal) ECG abnormalities such as ≥1 mm ST-elevation or horizontal ST-segment depression on ≥3 leads), and negative T-waves on rest ECG. The presence of at least one major or two minor criteria was considered diagnostic of rest toxicity. In patients with rest toxicity, EST was avoided during therapy. Patients without any sign or symptom at rest, or with only one minor criterion, underwent EST with Bruce protocol.

EST was considered positive if induced a new ST-segment elevation ≥2 mm, a horizontal or down-sloping ST-segment depression ≥1 mm in *>*3 consecutive leads, or the onset of frequent or complex VA or advanced atrioventricular block, even in the absence of typical symptoms.^19^ Additional tests were done in patients with suspected cardiotoxicity as clinically needed. When cardiotoxicity (at rest or after EST) was considered likely, capecitabine was withdrawn and each patient was treated according to the type and severity of the toxicity (eg, observation, hospital admission, or prescription of anti-ischemic or anti-arrhythmic drugs). If basal EST results were not available, the test was planned after 7 or more days off capecitabine and without any cardiovascular therapy. Cardiotoxicity was confirmed when EST without capecitabine (either done before therapy or after wash-out) was negative, ruling out false positives.

Patients without toxicity continued their planned therapy; a second stress test was planned for those undergoing 4-5 weeks continuous treatment; a final closing clinical evaluation was done 2 months after completion of the chemotherapy program. Before writing this article, we reviewed also the clinical charts of the patients who had been followed in our Institutions, checking for cardiovascular events and further chemotherapies after the study.

### Statistical Analysis

The chi-square test was used to assess the association of cardiotoxicity with age (<55 vs. ≥55 years) sex, family or personal history of IHD, and CVRF. Odds ratios (ORs) and their corresponding 95% confidence intervals (CIs) were computed using unconditional logistic regression models. SAS software version 9.4 (SAS Institute, Cary, USA) was used. Statistical tests were 2-sided. A *P-*value <.05 was considered statistically significant.

## Results

**Figure 1.**
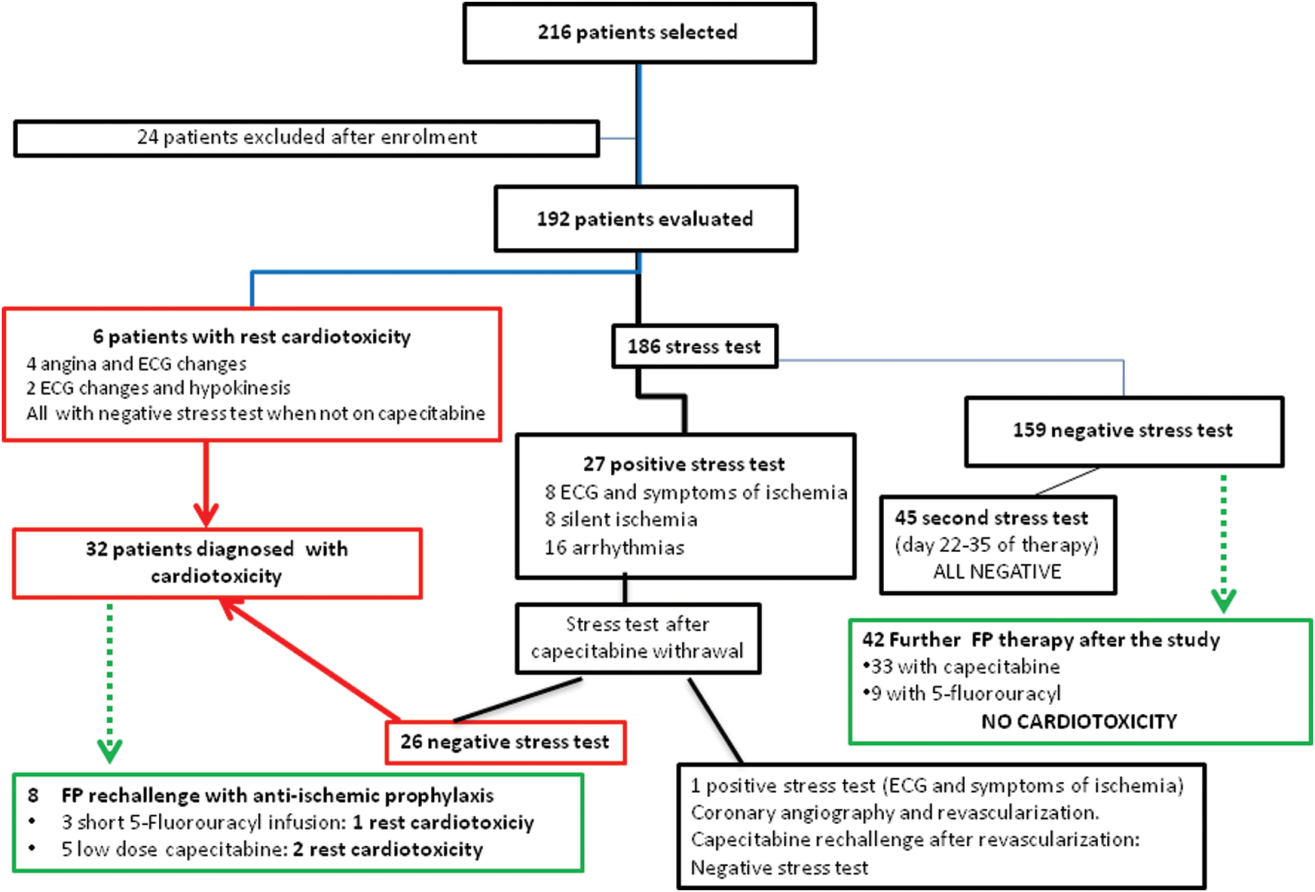
Summary of the study results. FP, fluoropyrimidines.

A total of 216 patients fulfilled the inclusion criteria (Fig. 1). Of these, 24 patients were excluded after enrolment (22 had switched to alternative chemotherapy regimens and two withdrew consent). The study population, therefore, consisted of 192 patients (Table 1), including 11 with a history of IHD (three with previous myocardial infarction) and 136 with one or more CVRF. Seventy-three patients were on chronic therapy with one or more cardiovascular drugs (mostly diuretics and beta-blockers); there were no patients taking nitrates (Table 1). Most patients had a gastrointestinal cancer (120 rectal carcinomas) while 19 had breast cancer.

**Table 1.** Baseline clinical characteristics of the 192 patients enrolled in the study.

Characteristic	All	Toxicity	No toxicity
Age, years, median, mean (SD)	63, 62 (+11)	61, 62 (+9)	62, 63 (+11)
Sex, *n* (%)			
Male	115 (59.9)	24 (75)	91 (57)
Female	77 (40.1)	8 (25)	69 (43)
Tumor, *n* (%)			
Gastrointestinal cancer	173 (90)		
Rectum	120 (62.5)	21 (65.6)	99 (61.9)
Gastroesophageal	26 (13.5)	7(21.9)	19 (11.9)
Upper bowel, liver, pancreas	8(4.1)	4(12.5)	4 (2.5)
Breast cancer	19 (10)	0	19 (11.9)
Family history of ischaemic heart disease, *n* (%)	24 (12.5)	5(15.6)	19 (11.9)
Body mass index, *n* (%)			
20-27 (normal)	153 (79.6)	26 (81.2)	134 (83.8)
28-31(overweight)	25(13)	5(15.6)	20(12.5)
>32 (obese)	7 (3.6)	1(3.1)	6 (3.8)
Diabetes, *n* (%)	15 (7.8)	3(9.3)	12(7.5)
Treated with diet alone	4 (2)	0	4 (2.5)
Treated with oral hypoglycaemic agents	8 (4.1)	3(9.3)	5 (3.1)
Treated with insulin	3(1.6)	0	3 (1.9)
Smoking habit, *n* (%)			
Never	122 (62)	21 (65.6)	101 (63.1)
Current, up to 10 cigarettes/day	12 (6.3)	1(3.1)	11 (6.9)
Current, 11-20 cigarettes/day	17 (8.9)	2(6.2)	15 (9.4)
Current, >20 cigarettes/day	6 (3.1)	1(3.1)	5 (3.1)
Former	35 (18.2)	7(21.9)	28 (17.5)
Hypertension,^a^*n* (%)	73 (38)	14 (43.8)	59 (36.9)
Mild	30 (15.6)	3(9.3)	27 (16.9)
Moderate	40 (20.8)	11(34.4)	29 (18.1)
Severe	3 (1.6)	0	3(1.9)
Blood cholesterol,^b^*n* (%)			
Normal	140 (72.9)	21(65.6)	119 (74.4)
Mildly elevated	37 (32.3)	9 (28.1)	28(17.5)
Moderately elevated	13(6.8)	2(6.2)	11(6.9)
Severely elevated	2 (1)	0	2 (1.2)
Cardiovascular risk factors (active smoking, overweight, diabetes, hypercholesterolemia, hypertension), *n* (%)			
None	56 (29.2)	7(21.9)	50 (31.3)
One	62 (32.3)	10 (31.2)	52 (32.5)
Two	52 (27.1)	9 (28.1)	43 (26.9)
Three	16 (8.3)	4(12.5)	12 (7.5)
More than three	6 (3.1)	2(6.2)	3(1.9)
History of ischaemic heart disease, *n* (%)	11 (5.7)	4(12.5)	7 (4.4)
On medical therapy	4 (2)	2(6.2)	2 (1.25)
Previous revascularization	4 (2)	1(3.1)	3(1.9)
Previous myocardial infarction	3 (1.6)	1(3.1)	2(1.25)
Ongoing therapies, *n* (%)			
Cardiovascular drug ^c^	73 (38)	11 (34.4)	60 (37.5)
Beta-blockers	35(18.2)	8(25)	27 (16.9)
Calcium channel blockers	12(6.3)	2(6.2)	10 (6.3)
Ace-inhibitors/Angiotensin Receptor’s blockers	19(10)	5(15.6)	14 (8.8)
Diuretics	56 (29.2)	11(34.4)	45(28.1)
Radiotherapy	120 (62.5)	25(78.1)	95 (59.4)
Oxaliplatin	26 (13.5)	7(21.9)	18 (11.3)
Docetaxel plus oxaliplatin	5 (2.6)	1(3.1)	4(2.5)
Epidoxorubicin plus oxaliplatin	7 (3.6)	2(6.2)	5(3.1)
Mitomycin	8 (4.1)	0	8 (5)
Irinotecan	4 (2)	0	4(2.5)
Bevacizumab	3 (1.6)	0	3(1.9)
Vinorelbine	7 (3.6)	0	7 (4.4)

Hypertension was defined as mild if maintained within the normal range with a single drug, moderate if required two drugs, severe if it required 3 or more drugs.

Blood cholesterol was considered normal if <200 mg/dL without therapy, mildly elevated if 201-240 mg/dL without therapy or <200mg/dL with diet, moderately elevated if requiring low-moderated dose statin therapy; severely elevated if requiring high dose statin therapy and possibly ezetimibe.

There were no patients on nitrates. Lipid-lowering therapies and aspirin were not tabulated.

Capecitabine doses ranged between 1000 and 3000 mg/m^2^·day (median, 1605 mg/m^2^·day). Treatment was administered either daily (for 2 weeks every 3 weeks for 6-8 courses in 21 patients or for 4 weeks in 159 cases) or Monday to Friday (the days of RT) for 5 weeks (12 cases). The 120 patients with rectal carcinoma received 4 or 5 weeks of capecitabine plus local radiotherapy.

### Study Outcomes

**Figure 2.**
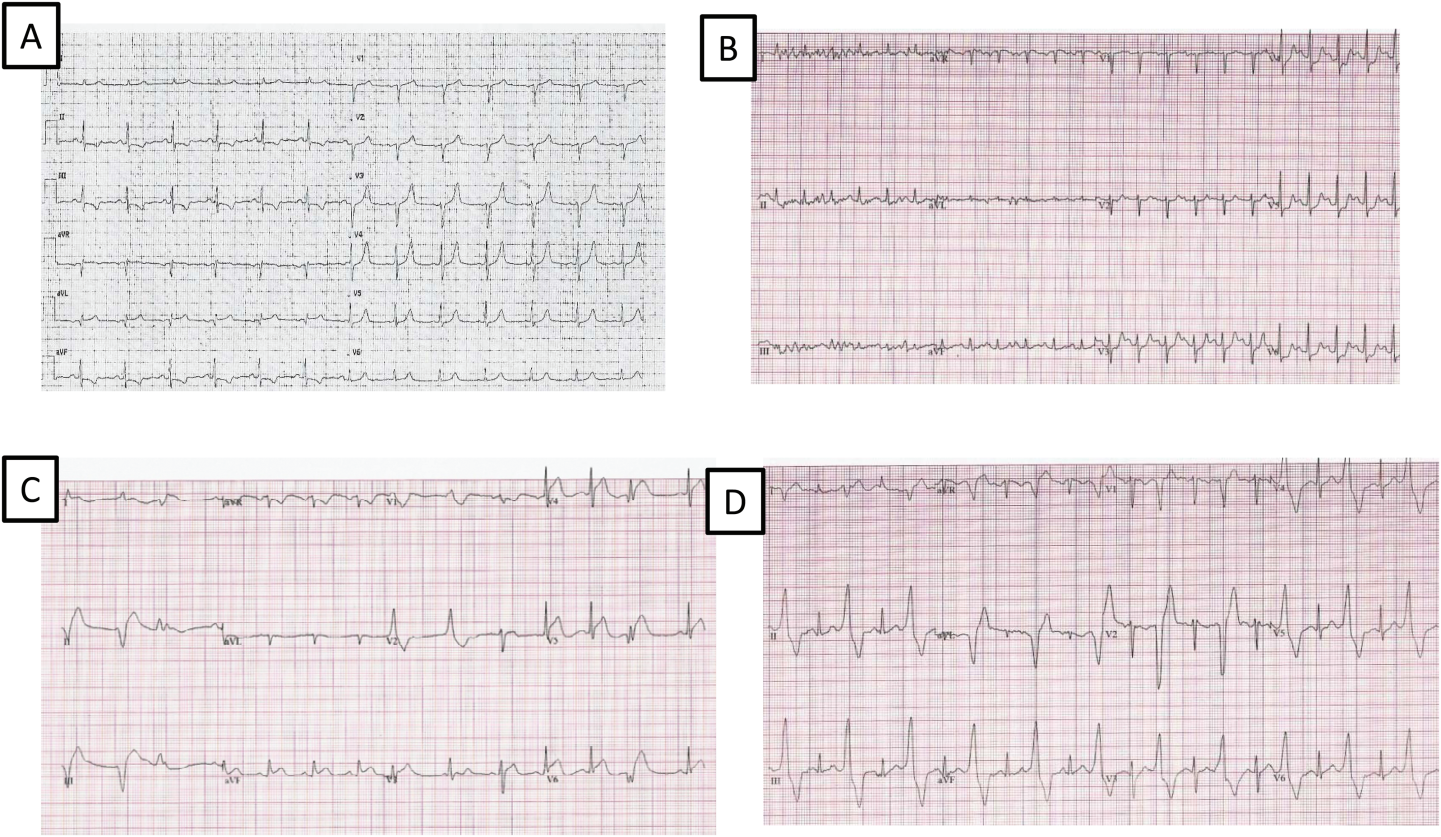
Different ECG aspects: (A) Male 58: rest ECG signs of ischemia. The echocardiogram showed a diffuse hypokinesia with reduced LV function. (B) Male, 59: ST-segment depression during stress test. (C) Male, 49 with ST segment elevation on D3, aVF, V3-V6, and ST-segment depression on V1 during stress test; tracing at the tenth minute of the recovery phase, showing persistent ST segment elevation and ventricular arrhythmias. (D) Female, 46: ventricular arrhythmias during stress test.

There were no deaths, and all patients completed at least one EST. Six patients (3%) had ischemic ECG changes at rest (Table 2, Fig. 2A), with four of them reporting typical angina (one was admitted to a different hospital with the acute coronary syndrome) and two demonstrating diffuse myocardial hypokinesia on 2DE. These patients all had a negative EST off capecitabine (one immediately before starting therapy, five after capecitabine withdrawal).

**Table 2.** Characteristics of 6 patients with cardiotoxicity at rest.

Sex	Age, years	Cardiovascular risk factors	Cancer	Timing^a^	Symptoms	ECG abnormalities	Echocardiogram abnormalities	Troponin^b^	Coronary angiography
F	62	Diabetes, hypertension, former smoker^c^	Rectal	3	Typical angina	Diffuse ST-segment depression	No changes	Negative	ND
M	59	Dyslipidaemia	Rectal	4	Typical angina	ST-segment elevation	ND	Negative	Yes^d^
M	59	None	Gastric	13	None	Negative T-waves	Diffuse hypokinesia, EF 45%	Negative	ND
M	68	Diabetes, hypertension, history of angina^e^	Rectal	4	None	ST-segment depression	Diffuse hypokinesia, EF 46%	Negative	ND
F	56	None	Colon	9	Typical angina	Negative T-waves	ND	ND	ND
F	57	None	Gastric	28	Chest pain	Negative T-waves, prolonged QT, ST-segment depression	EF drop from 73% to 65%	ND	ND

Timing defined as day of therapy during cycle when toxicity was detected.

Troponin was considered negative if within the normal limit of the laboratory.

Stopped smoking 6 years ago.

Coronary angiography, done urgently at a different hospital, detected a 60% stenosis of the 1st diagonal vessel deemed not worthing revascularization.

This patient had a negative stress test before starting capecitabine.

Abbreviations: EF, ejection fraction; ND, not done.

The other 186 patients underwent EST on therapy days 10-38 (median 15), and an abnormal (positive) result was found in 27 (Fig. 1). Repeating EST after withdrawing capecitabine gave a positive result in only one patient, who underwent coronary angiography and percutaneous intervention on a critical stenosis of the right coronary artery. A subsequent EST during capecitabine rechallenge was negative; we concluded that in this patient the positive EST was due to coronary artery disease rather than to capecitabine and she was excluded by the cardiotoxicity group. Forty-five patients (on therapy for 4-5 weeks), with negative EST after 10-14 days of therapy, had a second EST on day 22-35, and it was negative in all.

Overall, exercise-induced cardiotoxicity was diagnosed in 26 patients (13.5% of the study group), including 21 men and five women (Table 3). ST-segment changes were observed in 15 patients (57.7%): elevation alone in 4, depression alone in 6, and both in 5 (Figure 2B and C). Sixteen patients had major arrhythmias: 1 had supraventricular tachycardia, 14 had frequent VA (including 5 cases with ventricular tachycardia, 1 with couplets, 1 bigeminal rhythm), and 1 patient developed a complete heart block shortly after the EST, requiring pacemaker implantation. Pacemaker interrogation documented the absence of recurrent heart block after withdrawing capecitabine.

**Table 3.** Patients with cardiotoxicity after stress test (Bruce protocol). Symptoms at rest and during effort, ECG changes, METs attained when the ECG abnormalities appeared and maximum METs attained in the stress test without capecitabine. One patient, who had a complete A-V block after the stress test and a pacemaker implanted, did not repeat the stress test (see text).

Sex	Age	During treatment with capecitabine						Without capecitabine	
		Symptoms at rest	Symptoms at stress test	ST-changes at stress test	Number of ECG leads with ST-abnormalities	Arrhythmias	METs^a^	METs^b^	Heart rate (%)
M	62	Atypical	Palpitations			Ventricular couplets	4	10.5	86
M	47	Epigastric pain	Sore throat	4 mm ST elevation, 3 mm ST depression	7		4	12	100
M	47	No	No	2 mm ST elevation; 1 mm ST depression	8		8	10.5	95
M	55	No	No			Frequent VEB	7	12	85
M	65		No	2 mm ST depression	6		4	5,4	80
M	63	No	Angina	2 mm ST depression	1		6.3	6.5	99
M	74	No	No			SVT runs	4	5.8	86
M	75	No	No			Frequent VEB	7	5	90
M	74	No	Syncope (after stress test)			Complete A-V block after stress test	5.6	Not done (after pacemaker implantation)	
F	75	No	No	2 mm ST depression	3		7	7	94
M	68	Atypical CP	No	3 mm ST depression	4	NSVT	4.6	6.8	92
M	61	No	No			Frequent VEB	4	12	72
M	49	Atypical CP	Atypical	5 mm ST elevation	9	NSVT	4.6	13.2	91
M	60	No	No	1 mm ST depression	4		6	10	87
M	65	No	No	3 mm ST elevation, 1 mm ST depression	6	Frequent VEB	5.6	10	86
F	61	Epigastric pain	Typical angina	2 mm ST elevation	6	Frequent VEB	4	4.6	93
M	67	No	No			Frequent VEB	4	6	82
M	71	No	No			NSVT	4	10	91
M	60	No	No	4 mm ST elevation	9		7	10.7	86
M	59	No	No	3 mm ST depression	3		7	10	99
F	79	No	Atypical			NSVT and frequent SVA	6	7	72
F	46	No	No			Frequent VEB	7	10.7	90
M	42	Effort angina	Typical angina	2 mm ST elevation, 1 mm ST depression	5		4	13.5	86
M	68	No	Chest pain	3 mm ST elevation, 3 mm ST depression	6		7	10.3	80
M	75	No	No			NSVT	4.6	7	91
F	56	Atypical CP	Atypical, dizziness	4 mm ST elevation	5	Frequent VEB	7	13	87

METs attained at the time of appearance of clinical or ECG abnormalities (test during capecitabine).

METs attained at the peak of effort (test without capecitabine).

Abbreviations: Heart rate, heart rate at peak stress expressed at the % of the target heart rate according to age; A-V, atrio-ventricular; CP, chest pain; METs, Metabolic Equivalent of Task; SVA, supraventricular arrhythmias; SVT, supraventricular tachycardia; VEB, ventricular ectopic beats (defined as “frequent” if more than 10% of all ventricular depolarizations during any 30-s ECG recording); NSVT, ventricular tachycardia (*>*3 VEB).

**Figure 3.**
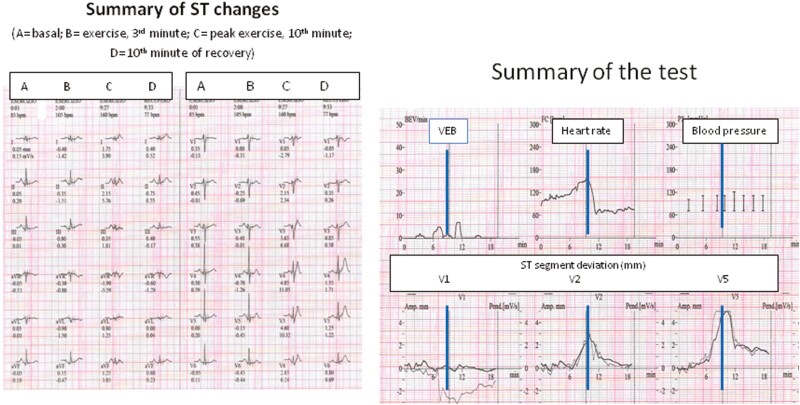
Female, 55, triathlon runner, on capecitabine for 3 weeks. Asymptomatic ST-segment elevation on V2-V5 at peak exercise. *Left*: representative traces. *Right*: trend of ventricular ectopic beats (VEB), heart rate, blood pressure, ST segment deviation during exercise and recovery. Blue line: end of exercising.

In 5 patients, either ST-segment elevation or VA appeared during the recovery phase, not having been seen during effort; in 8 patients, the abnormalities that had appeared during the test worsened in the recovery phase (Figs. 2D and 3).

Altogether, 32 patients (aged 42-75, mean and median 61) experienced cardiotoxicity either at rest or during effort (16.7%). The clinical presentation was with typical angina in 8 (25%) of them and atypical symptoms in 7 (22%); 17 (53%) had no symptoms at all. As regards the ECG findings, 16 patients (50%) had arrhythmias and 6 (19%) had pathological ECG findings at rest.

### Additional Information from the Extended Follow-up

In 150 out of 192 patients a follow-up after the study was available; 30 of them pertained to the cardiotoxicity group. One patient (with already known IHD) in the cardiotoxicity group and 1 in the group without cardiotoxicity had an acute myocardial infarction, 2 and 4 years after participating in the study, respectively; the others did not have any cardiovascular event, including the patient with acute coronary syndrome undergoing coronary angiography, who survived 5 more years and eventually died of cancer progression.

Months or years after the study, 50 patients (42 without and 8 with cardiotoxicity during the study) required further chemotherapy with FP and were screened with regular rest ECG and clinical evaluations during the rechallenge. The group of 42 without cardiotoxicity received 6-12 courses of full dose regimens (33 with capecitabine, 9 with five-days 5-FU infusions): cardiotoxicity was never observed. To protect the 8 patients with the previous cardiotoxicity, during rechallenge we: (1) used modified schedules (5 with reduced capecitabine dose, 3 with short 5FU infusion); (2) associated anti-ischemic therapy; (3) advised patients to avoid physical effort. With this approach, three patients had rest toxicity (two with capecitabine, one with 5FU) so FP was definitely interrupted; the other 5 tolerated the rechallenge (Fig. 1).

### Risk Factors for Capecitabine Cardiotoxicity

Age, sex, CVRF, or family or personal history of IHD were not predictive of cardiotoxicity both at multivariate and univariate analysis (Table 4, Supplementary Table 1). In contrast, patients who had symptoms consistent with angina equivalents or even more atypical symptoms (epigastric pain, malaise, dizziness) during daily life before the planned visit with stress test were 20.78 times more likely to have cardiotoxicity (logistic regression OR = 20.78, 95% CI, 3.37-50.04, *P* <.001). Surprisingly, patients who received capecitabine 5 days/week had a higher likelihood of cardiotoxicity than those on continuous daily treatment (OR = 13.68, 95% CI, 5.73-75.42, *P* <.001).

**Table 4.** Odds ratios for toxicity and corresponding 95%CI, according to clinical parameters in multiple logistic regression including terms for age, days/week of treatment, and symptoms.

Variable	OR (95%CI)	*P*-value
Weekly days of treatment, *n*		
7	1	
5	13.68 (3.37-50.04)	<0.01
Symptoms during daily life		
None	1	
Yes (either typical or atypical)	20.78 (5.73-75.42)	<0.01

## Discussion

### Incidence of Capecitabine Cardiotoxicity

This study indicates that nearly 1 in 5 patients on capecitabine shows major arrhythmias and/or myocardial ischemia when followed with an active surveillance protocol. Most of the cases of cardiotoxicity were identified during EST, highlighting a risk burden previously not recognized and yet present in patients on capecitabine therapy.

Reported rates of FP cardiotoxicity range widely according to differences in study design.^5,8,15,19-27^ Retrospective studies (based on symptoms reported in the clinical charts) report an incidence of 4.3-7%.^13,21,24-26^ Two prospective studies based on symptoms found 5.5-6.5% of capecitabine cardiotoxicity,^9,10^ while other prospective studies including ECG found rates of 8.5-35% fluoropyrimidine cardiotoxicity.^8,14-16,23,27,28^ Some of these studies, however, included in the term “cardiotoxicity” ECG changes of little, if any, clinical significance, as asymptomatic sinus bradycardia or PR and QT changes.^14,15,28^ A recent prospective study including Holter monitoring during 5FU infusion found 14% of ischemic changes at Holter, with 5.6% cases of acute coronary syndromes and 1.8% of symptomatic arrhythmias.^27^ In this study, 75% of ischemic changes were asymptomatic, but in 3 patients they preceded myocardial infarction or cardiac arrest. Of note, the same group found an incidence of 4-5% only in two retrospective studies based on symptoms and published in the past years.^21,24^

Capecitabine is a life-saving cancer treatment: to reliably balance the risks (severe or fatal cardiotoxic effects) and benefits of this therapy, both underdiagnosis and overdiagnosis of cardiotoxicity should be avoided. We used ECG and EST to avoid underdiagnosis of asymptomatic events and of events elicited by physical activity. We excluded sinus bradycardia, rare VA, minor ECG changes, anginal symptoms without ECG or echocardiographic abnormalities from the diagnostic criteria, to avoid overdiagnosis. Re-evaluation with EST off therapy increased the specificity of the diagnosis; according to the Naranjo criteria, adverse drug reactions are considered “likely” when they appear in a close temporal relationship with drug administration, they disappear after drug withdrawal, and no other possible causes are identified.^29^

### Clinical Presentation of Capecitabine Cardiotoxicity

More than half of the patients were completely asymptomatic, even when ischemic ECG changes or frequent VA were detected. This finding suggests that studies based on clinical symptoms may underestimate the problem. In the recent study of Dyhl-Polk^27^ half of the patients who later developed acute myocardial infarction (one with cardiac arrest) during or at the end of 5FU infusion had silent ischemia at Holter monitoring in the previous days of infusion. This observation corroborates the risk of silent ischemia. Atypical symptoms were also observed, some consistent with angina equivalents; these symptoms might be misinterpreted by non-cardiologists.^30^

FP-induced ischemia has classically been described as resting ST-segment elevation.^4,27,28,31^ In this study, only 1 of the patients with resting cardiotoxicity had ST-segment elevation, while the other 5 patients had ST-segment depression and/or negative T-waves. Of note, in 2 completely asymptomatic patients, resting cardiotoxicity was suspected from their ischemic ECG changes, corroborating the utility of routine ECG to screen for FP toxicity.^16^

EST results showing ST-segment depression are indicative of ischemia.^19^ Yet ST-segment elevation induced by physical effort is rare, but it has been described in patients with vasospastic angina and during FP treatment.^17,32,33^ In this study, ST-segment elevation and depression occurred at similar frequencies. Thus, probably the mechanism of FP-induced ischemia is not always vasospasm, which is usually characterized by ST-segment elevation.^34^

Among arrhythmias, VA was frequent in this study, as previously reported for patients on 5FU,^35-37^ but was not always accompanied by ECG signs of myocardial ischemia or QT interval prolongation, which is frequent during FP therapy.^38^ The appearance or worsening of ST-T changes and VA during the recovery phase of EST implies usually a worse prognosis.^39-41^ The significance of this behavior in the transient form of capecitabine cardiotoxicity merits further investigation.

### Exercise Stress Testing

EST was planned after at least 10 days of treatment to increase its sensitivity because FP cardiotoxicity is more frequent in prolonged therapies.^16,24,42^ We observed more cases of stress-induced cardiotoxicity than rest cardiotoxicity (26 vs. 6). In 12 cases the toxicity was evident after a workload of *<*4.6 Metabolic Equivalents of Task (METs), corresponding to activities as digging, making beds, walking upstairs, washing windows, mowing the lawn, painting a wall, bicycling at 10 km/h.^43^ Silent ischemia (frequent in our study) implies a worse prognosis in IHD^44^; it is most dangerous when effort related, because a subject without warning symptoms may continue (or even increase) his/her physical activity. Since some patients with cancer are rather young with good performance status, their risk of effort-induced cardiac events must be considered. The patients without cardiotoxicity at EST after *>*10 days completed even prolonged treatments safely and 42 of them tolerated new treatments when required. This finding suggests that EST has a negative predictive value for clinically relevant cardiotoxicity. The need for prospective evaluation using prespecified diagnostic criteria to understand the incidence of fluoropyrimidines has been recently highlighted.^11^ Integration of ECG and EST among the peri-chemotherapy interventions should help patient management and ensure patient safety.

### Predictors of Cardiotoxicity

Only 2 predictors of capecitabine cardiotoxicity emerged from our study. The presence of even atypical symptoms during daily life (confirming our previous study on 5FU),^16^ and the therapeutic schedule 5 days/week. The last result was unexpected (possibly due to a type 1 statistical error): treatment protocols with shorter FP exposures should be less toxic, by hindering capecitabine accumulation.^5,9,17,24^ These contradictory findings should be resolved in larger studies. Our study did not confirm some previously reported risks for cardiotoxicity, including age >55 years, female sex, use of platinum, hemoglobin levels, IHD, and common CVRF.^7,15,21,24,25,45-47^ The proportion of patients with IHD in this study was higher in the cardiotoxicity group, but the difference was not significant, possibly because of the small number of patients and this aspect should be investigated in a larger group.

### Study Limitations

The patients included in the study are only a part of all those treated with capecitabine in our institutions. The main difficulty in enrolling patients was to schedule EST in a short time window (between days 11 and 14 of therapy) for patients on treatment for 2 weeks every 3, because this required an additional hospital visit. Echocardiography during chemotherapy was performed only in patients with symptoms or ECG abnormalities, and 12-lead Holter monitoring to detect silent ischemia during daily life^27,28,48^ was not done; we may have missed some asymptomatic cardiotoxicities. The presence of previously undiagnosed IHD was excluded on a clinical basis; according to the guidelines, coronary angiography was limited to patients with a medium-high probability of coronary artery disease or positive EST after wash-out.^19,49^ It should be noted that, according to the follow-up data available, we did not miss any case of significant IHD.

## Conclusions

The incidence of clinically relevant capecitabine-induced cardiotoxicity in this study was higher than previously reported by most studies based on symptoms only, and many patients were asymptomatic or had atypical symptoms, confirming that this toxicity may be underestimated in clinical practice. Most cardiotoxic events occurred during physical effort and EST was instrumental in unmasking cardiotoxicity in these asymptomatic patients. Therefore, patients undergoing capecitabine therapy should be advised against moderate to severe physical effort and should promptly inform their physicians of new symptoms. Every new symptom or ECG abnormality should be carefully considered, even in young patients with a low probability of IHD. An active screening (including ECG and stress test) during the first course of therapy may identify a population at low risk of FP cardiotoxicity.

## Supplementary Material

oyab035_suppl_Supplementary_Table_S1

## Data Availability

The data underlying this article will be shared on reasonable request to the corresponding author.
