# Supplementary material for: Cardiotoxicity from Capecitabine Chemotherapy: Prospective Study of Incidence at Rest and During Physical Exercise
Source: Oncologist. 2022 Feb 16;27(2):e158–67. doi: 10.1093/oncolo/oyab035 (PMC8895550; doi:10.1093/oncolo/oyab035)
Supplement: oyab035_suppl_Supplementary_Table_S1 [file oyab035_suppl_supplementary_table_s1.pdf]

The Oncologist
The official journal of the Society for Translational Oncology

Supplemental Table for:
Cardiotoxicity from capecitabine chemotherapy: prospective study of incidence at rest and during physical exercise
Chiara Lestuzzi et al.

Supplementary Table 1 Odds ratios for toxicity and corresponding 95% confidence intervals, according to clinical parameters, adjusted for age in logistic regression

**Variable**		**ALL N**	**TOX N (%)**	**NO TOX N (%)**	**OR (95% CI)**	***P*-value**
Sex						
	Male	115	24 (75)	91(57)	1	
	Female	77	8 (25)	69 (43)	0.45 (0.19-1.05)	0.07
Tumour						
	Gastrointestinal cancer	173	32 (100)	141 (88)	1	
	Breast cancer	19	0	19 (12)	-	NC
Family history of ischemic heart disease						
	No	168	27(84)	141 (88)	1	
	Yes	24	5 (16)	19 (12)	1.46 (0.50-4.30)	0.49
Obesity						
	No	153	26 (81)	134 (84)	1	
	Overweight-obesity	39	6 (19)	26 (16)	1.14 (0.43-3.06)	0.79
Diabetes						
	No	177	29 (91)	148 (92)	1	
	Yes (any grade)	15	3 (9)	12 (8)	1.27 (0.34-4.78)	0.72
Current smoker						
	No	157	28 (87)	129 (81)	1	
	Yes	35	4 (13)	31 (19)	0.76 (0.27-2.16)	0.61
Blood pressure						
	Normal	119	18 (56)	101 (63)	1	
	Hypertension (any grade)	73	14 (44)	59 (37)	1.34 (0.60-2.98)	0.47
Blood cholesterol						
	Normal	140	21 (66)	119 (74)	1	
	Elevated (any grade)	52	11 (34)	41 (26)	1.51 (0.67-3.40)	0.32
Ischaemic heart disease						
	No	181	28 (88)	152 (95)	1	
	Yes	11	4 (12)	8 (5)	2.70 (0.76-9.61)	0.12
Number of cardiovascular risk factors						
	0	56	7 (22)	50 (41)	1	
	>0	136	25 (78)	110 (69)	1.56 (0.62-3.91)	0.34
Radiotherapy						
	No	72	7 (22)	64 (31)	1	
	Yes	120	25 (78)	96 (69)	1.65 (0.73-3.70)	0.23
Haemoglobin (mg%)						
	≥12	133	26 (81)	107 (67)	1	
	<12	59	6 (19)	53 (33)	0.46 (0.18-1.9)	0.11
Weekly days of treatment						

	7	180	25 (78)	155 (97)	1	
	5	12	7 (22)	5 (3)	9.44 (2.68-0.37)	<0.001
Symptoms before stress test						
	None	179	23 (72)	156 (98)	1	
	Any (typical or atypical)	13	9 (28)	4 (3)	15.70 (4.42-55.86)	<0.001

NC= not computable; TOX= patients with cardiotoxicity; NO TOX= patients without cardiotoxicity
